# Benzoic Acid and Its Hydroxylated Derivatives Suppress Early Blight of Tomato (*Alternaria solani*) via the Induction of Salicylic Acid Biosynthesis and Enzymatic and Nonenzymatic Antioxidant Defense Machinery

**DOI:** 10.3390/jof7080663

**Published:** 2021-08-16

**Authors:** Yasser Nehela, Naglaa A. Taha, Abdelnaser A. Elzaawely, Tran Dang Xuan, Mohammed A. Amin, Mohamed E. Ahmed, Asmaa El-Nagar

**Affiliations:** 1 Department of Agricultural Botany, Faculty of Agriculture, Tanta University, Tanta 31511, Egypt; elzaawely@agr.tanta.edu.eg (A.A.E.); asmaa.elnagar@agr.tanta.edu.eg (A.E.-N.); 2 Citrus Research and Education Center, Department of Plant Pathology, University of Florida, 700 Experiment Station Rd., Lake Alfred, FL 33850, USA; 3 Vegetable Diseases Research Department, Agricultural Research Center, Plant Pathology Research Institute, Giza 12619, Egypt; naglaa_abdelbaset@yahoo.com; 4 Graduate School for International Development and Cooperation, Hiroshima University, Hiroshima 739-8529, Japan; tdxuan@hiroshima-u.ac.jp; 5 Department of Chemistry, College of Science, Taif University, P.O. Box 11099, Taif 21944, Saudi Arabia; mohamed@tu.edu.sa; 6 Department of Horticulture, Faculty of Agriculture, Tanta University, Tanta 31511, Egypt; dr.memahmed@agr.tanta.edu.eg

**Keywords:** *Alternaria*, early blight, tomato, benzoic acid, ρ-hydroxybenzoic acid, protocatechuic acid, salicylic acid, reactive oxygen species (ROS), antioxidant

## Abstract

Tomato early blight, caused by *Alternaria solani*, is a destructive foliar fungal disease. Herein, the potential defensive roles of benzoic acid (BA) and two of its hydroxylated derivatives, *ρ*-hydroxybenzoic acid (HBA), and protocatechuic acid (PCA) against *A. solani* were investigated. All tested compounds showed strong dose-dependent fungistatic activity against *A. solani* and significantly reduced the disease development. Benzoic acid, and its hydroxylated derivatives, enhanced vegetative growth and yield traits. Moreover, BA and its derivatives induce the activation of enzymatic (*POX*, *PPO*, *CAT*, *SlAPX*s, and *SlSODs*) and non-enzymatic (phenolics, flavonoids, and carotenoids) antioxidant defense machinery to maintain reactive oxygen species (ROS) homeostasis within infected leaves. Additionally, BA and its hydroxylated derivatives induce the accumulation of salicylic acid (SA) and its biosynthetic genes including isochorismate synthase (*SlICS*), aldehyde oxidases (*SlAO1* and *SlAO2*), and phenylalanine ammonia-lyases (*SlPAL1*, *SlPAL2*, *SlPAL3*, *SlPAL5*, and *SlPAL6*). Higher SA levels were associated with upregulation of pathogenesis-related proteins (*SlPR-1*, *SlPR1a2*, *SlPRB1-2*, *SlPR4*, *SlPR5*, *SlPR6*), nonexpressor of pathogenesis-related protein 1 (*SlNPR1*), and salicylic acid-binding protein (*SlSABP2*). These findings outline the potential application of BA and its hydroxylated derivatives as a sustainable alternative control strategy for early blight disease and also deciphering the physiological and biochemical mechanisms behind their protective role.

## 1. Introduction

Tomato (*Solanum lycopersicum* L., family *Solanaceae*) is one of the world’s leading vegetable crops [1]. It is grown in over 170 countries, with the total productivity of 180.77 million tonnes, produced from approximately five million hectares [2]. In Egypt, the total yield of tomatoes in 2019 was 6,751,856 tonnes and the harvested area was 173,276 hectares [2]. Tomato is highly susceptible to numerous fungal, oomycetes, bacterial, and viral phytopathogens that significantly reduce the crop quality and yield quantity [3,4,5,6].

Early blight, caused by *Alternaria solani* (Ellis et G. Martin) Sorauer (Ascomycota, Pleosporaceae), is one of the most destructive foliar diseases of tomato that decreasing its production by 80% [7]. The typical disease symptoms initially form on older foliage near the ground as small dark-colored lesions that might expand to half-inch (3–12 mm) in diameter. Larger spots are round, brown, and have distinctive target-like concentric rings [8,9]. In severe infection cases, leaves turn brown, died, and fall off, or they might cling to the stem. The epidemic of early blight disease is widely recorded in regions with higher humidity, rainfall, and temperatures [10]. Tomato’s early blight disease can destroy all aerial parts of the tomato plant including the shoot system, fruit, and at all growing stages. In addition, *A. solani* inhibits the photosynthesis rate in infected leaves by inhibiting photo-system II activity and decreasing the chlorophyll content [11,12]. The degradation of photosynthetic pigments caused by *A. solani*, causing a significant reduction in growth and thus a decrease in yield [12].

Integrated pest management (IPM) strategies of early blight on tomatoes include growing resistant cultivars, maintaining plant vigor, crop rotation, nutrition management, sanitation, grafting, but mainly rely on using chemical fungicides. However, fungicides are expensive, environmentally harmful, and negatively affect non-target plants and animals. Moreover, regular fungicide application can lead to the development of fungicide resistance in pathogen populations [13]. In response to these concerns, researching effective alternative control strategies to conventional fungicides has become one of the top priorities in the agrochemicals industry. The alternative control strategies including the development and implementation of sustainable eco-friendly natural compounds to reduce the use of fungicides partially or entirely. Natural compounds are also used as a basis for the production of new pesticides and the finding of novel target sites for pesticides [14]. Phenolic compounds may be one of the promising alternative strategies.

Phenolic compounds are a cluster of secondary metabolites that contain at least one phenol unit with one or more hydroxyl groups [15]. Phenolics are derived from _L_-phenylalanine via the shikimic acid and phenylpropanoid pathways [16]. They are ubiquitously distributed phytochemicals found in most plant tissues and play a vital role in plant response to biotic and abiotic stresses, particularly pathogens infection [16,17]. Phenolic compounds are often developed and accumulated in the sub-epidermal layers of plant tissues exposed to different abiotic and biotic stresses [18]. Phenolic compounds are also known as strong antioxidants that can scavenge free radicals [19].

Benzoic acid (BA) and its hydroxylated derivatives, *ρ*-hydroxybenzoic acid (HBA) and protocatechuic acid (PCA), are among the most common phenolic compounds. Benzoic acid is an organic aromatic monocarboxylic acid that possesses potential antibacterial and antifungal activities [20], as well as protective roles against various environmental stresses such as heat, drought, and chilling stress [21,22]. For instance, BA and 57 of its derivatives showed potential antifungal properties against the phytopathogenic fungus *Eutypa lata*, the causal agent of dieback disease, and defined a possible structure-activity relationship [23]. Additionally, BA reduced the growth rate of *A. citri* and *A. alternata* the casual agents of stem-end rot and internal core rot (black rot) in citrus fruits under laboratory conditions [24]. Likewise, it reduced the disease severity of Alternaria blight, caused by *Alternaria brassicae* and *Alternaria brassicicola*, on leaves and pods of Indian mustard under field conditions [25]. Moreover, BA has an inhibitory effect on some soil-borne fungi, such as *Fusarium* sp. [26], and seed-borne pathogens such as *Aspergillus flavus*, *Penicilliun citrinum*, and *Alternaria* *alternata* [27].

On the other hand, the pharmacological activities of both HBA and PCA were well-reported previously. For example, HBA, intercalated into layered double hydroxides (LDHs, with Zn:Al molar ratio = 4:1), effectively possessed antifungal properties against *Candida albicans*, and a strong antibacterial effect against *Staphylococcus aureus* [28]. Similarly, PCA showed promising multidirectional pharmacological activities that included anti-inflammatory, antioxidative, antimicrobial, antibacterial, and antiviral properties [29]. In plants, the potential roles of HBA and PCA in enhancing the plant resilience to biotic and abiotic stresses are inadequately studied. For instance, limited studies reported that exogenous HBA mitigated the heat tolerance in cucumber leaves via the regulation of antioxidant enzyme activity [30]. Besides, PCA has demonstrated strong antifungal activity against *Botrytis cinerea* and *Rhizoctonia solani.* However, the antifungal activity was not sufficient to inhibit the mycelial growth of soil-borne fungi *Fusarium oxysporum* and *Phytophthora capsici* [31]. Nevertheless, the physiological and biochemical mechanisms behind the protective role of these compounds are poorly understood.

Herein, the in vivo antifungal activities of BA and two of its hydroxylated derivatives (HBA and PCA) against *A. solani*, the causal agent of early blight of tomato were investigated. Moreover, we used integrative biochemical, metabolomic, and transcriptomic approaches to (i) explore the potential roles of BA and its hydroxylated derivatives in inducing tomato response(s) to fungal infection; (ii) elucidate the regulatory role of BA and its hydroxylated derivatives in mitigating the infection-associated antioxidant enzyme activities; (iii) understand the BA-mediated defensive response and its relationship with the major defense-associated phytohormone, salicylic acid, and its biosynthetic genes. We believe that BA and its hydroxylated derivatives are not only involved in buffering the antioxidant capacities of tomato plants upon fungal infection but also might be involved in the induction of the SA-mediated pathway to enhances the disease resistance against necrotrophic phytopathogens. BA and its hydroxylated derivatives might be a novel therapeutic strategy to mitigate the early blight on tomatoes.

## 2. Materials and Methods

### 2.1. In Vitro Antifungal Activity of Benzoic Acid and Its Hydroxylated Derivatives

#### 2.1.1. Tested Compounds

**Figure 1:**
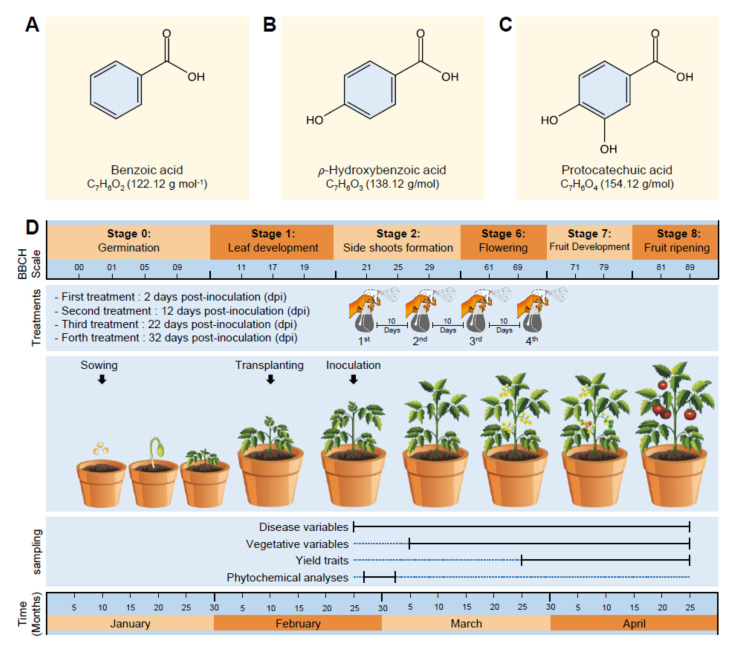
Chemicals and experimental design used in this study. (**A–C**) Chemical structure of benzoic acid (BA), *ρ*-hydroxybenzoic acid (HBA), and protocatechuic acid (PCA), respectively. Molecular weight/molar mass (g·mol^−1^) is mentioned between parentheses under the chemical formula of each compound. (**D**) Experimental design, application times, and sampling points used in this study. Growth development stages are corresponding to the phenological growth stages on BBCH-scale [32,33].

Benzoic acid (BA; Figure 1A) and two of its hydroxylated derivatives, ρ-hydroxybenzoic acid (HBA; Figure 1B), and protocatechuic acid (PCA; Figure 1C) were purchased from Sigma-Aldrich (Darmstadt, Germany). All tested compounds, in addition to the commercial fungicide Score, 25% (difenoconazole 25 EC), were firstly dissolved in 2 mL 100% dimethyl sulfoxide (DMSO) and then adjusted to a final volume of 100 mL using sterilized water to make a 1000 ppm stock solution (approximately 8.19, 7.24, and 6.49 mM of BA, HBA, and PCA, respectively) that has been diluted and used in all further experiments.

#### 2.1.2. Antifungal Activity

In vitro antifungal activities of BA, HBA, and PCA were assessed using the agar diffusion technique [34]. The experiment was organized in a completely randomized design, with six biological replicates. Briefly, gradient serial dilutions of each compound, as well as the commercial fungicide, were prepared by mixing appropriate volumes of each compound with 20 mL of the potato dextrose agar (PDA) medium in a sterilized Petri dish to obtain five final concentrations at 20, 40, 60, 80, and 100 ppm. The commercial fungicide Score, 25% (difenoconazole 25 EC) was used as a positive control, while sterilized DMSO, at a final concentration of 0.2% in the PDA medium, was used as a negative control. Subsequently, a 5 mm diameter mycelial plug of a freshly prepared *A. solani* culture was placed on the surface of the preprepared Petri dishes, then incubated at 27 ± 1 °C for seven days, until the mycelial growth covered the whole control plates. After 7 days, mycelium growth (mm) was recorded from each Petri dish. The growth inhibition percentage has been calculated using the following equation (1):

(1) $$\mathrm{Inhibition}\;\left(\%\right)=\frac{C\;-T}{C}\times 100$$

where “C” is mycelium growth (mm) in the negative control plate and “T” is mycelium growth (mm) in the treatment.

#### 2.1.3. Half-Maximal Inhibitory Concentration (IC_50_)

Probit regression analysis (PRA) was used to calculate the half-maximal inhibitory concentration (IC_50_) and IC_99_. Briefly, serial concentrations (0, 20, 40, 60, 80, and 100 ppm) of BA, HBA, and PCA were tested as described above and PRA was used to fit the probit/logit sigmoid dose-response curves and to calculate different inhibitory concentrations (IC_50_ and IC_99_) with 95% confidence intervals [35].

### 2.2. In Vivo Experiment

#### 2.2.1. Plant Materials and Growth Conditions

Throughout the study and in all experiments, the early blight-susceptible tomato genotype (*Solanum lycopericum* L.—Super strain B F_1_ hybrid) was used as an experimental plant. Seeds were collected from the Research Department of Vegetable Diseases, Institute of Plant Pathology Research (IPPR), Agricultural Research Center (ARC), Egypt, and sown in seedling trays (209 cells) for one month in the nursery. Seedling trays were placed under greenhouse conditions (27 ± 2 °C, 75 ± 5% RH, and 16:8 h L/D photo cycle) at Vegetable Diseases Research Department (VDRD), ARC, Sakha Agricultural Research Station, Kafr El-Sheikh, Egypt (31.094059° N, 30.933899° E). After 30 days from sowing and during the leaf development stage on BBCH-scale (Figure 1D) [34,35], seedlings were transplanted into plastic pots (30 cm in diameter) filled with sterilized clay soil and preserved under the same conditions as described above. All other horticultural practices were conducted as recommended. In all greenhouse experiments, the used tomato plants were healthy with no symptoms of early blight disease at the beginning of each experiment.

#### 2.2.2. Infection with *A. solani* and Treatment with Benzoic Acid and Its Derivatives

The germ tubes of A. solant can penetrate the leaf epidermis directly or enter through stomata. Two weeks after transplanting and approximately corresponding to the phenological growth stage 21 on BBCH-scale (Figure 1D), tomato seedlings were sprayed with a conidial suspension of a 7-days-old culture of *A. solani* (5 × 10^5^ conidia mL^−1^ prepared in sterilized water) using a manual one-gallon atomizer multipurpose pump sprayer (operating pressure between 40 and 60 PSI and a flow rate of 0.45 GPM).

#### 2.2.3. Treatment with BA, HBA, or PCA

Infected tomato plants were sprayed with 30 mL plant^−1^ of a 100-ppm solution of one of the tested phenolic compounds (BA, HBA, or PCA) 24 h post-inoculation (hpi). Besides, the commercial fungicide Score, 25% (difenoconazole 25 EC at the recommended dose) was used as a positive control, while sterilized water containing 0.2% DMSO was used as a negative control (henceforth Mock control). Tomato plants were treated via a foliar application using the same manual pump sprayer described above. Foliar application of phenolic compounds was repeated four times throughout the season, with 10 days interval between sprays. The whole experiment was carried out using a completely randomized design and repeated twice (six biological replicates per treatment), each replicate consists of 6 pots (one plant per pot) through two seasons (2020 and 2021) under greenhouse conditions as described previously. The treated plants were assessed for disease severity (during the first week after the first spray, and then every 10 days till the end of the experiment), vegetative growth characteristics (from the inoculation day and every 10 days till the end of the experiment), and yield traits (starting from the flowering stage and every 10 days till the end of the experiment. For leaf sampling, three leaves (2nd, 3rd, and 4th leaves) were collected from each plant at 0, 24, 48, 72, 96, and 120 h post-treatment (hpt).

#### 2.2.4. Disease Assessments

Disease severity (DS) of early blight was evaluated daily during the first week after inoculation, and then every 10 days till the end of the experiment. Disease severity was assessed based on the five-point (0–5) scale, as the percentage of leaf area covered by necrotic lesions [36]. Moreover, the area under the disease-progress curve (AUDPC) was calculated as described by [37].

#### 2.2.5. Vegetative Growth and Yield Assessment

Plant height (cm), number of leaves per plant, and total leaf area per plant (cm^2^) were assessed for all treatments (BA, HBA, and PCA) and both controls (fungicide and mock) every 10 dpt throughout the experiment. Likewise, shoots’ fresh and dry weights per plant were assessed at 60 dpt through drying in an oven at 70 °C for 72 h; the shoots’ dry weight was recorded. For yield traits, the average number of flowers per plant was assessed at 30, 40, 50, and 60 dpt. Moreover, the average number and weight of fruits, of each plant were also recorded and the total fruit yield per plant was calculated.

### 2.3. Phytochemical Analyses

#### 2.3.1. Photosynthetic Pigments

Chlorophyll *a*, chlorophyll *b*, and total carotenoids contents were determined using spectrophotometric methods as described by Dere et al. [38]. Briefly, approximately 100 ± 5 mg of fresh leaves were cut into small pieces and immersed for 24 h in 20 mL methanol (96%) at 4 °C and then filtered through Whatman 47 mm GF/C filter paper. The absorbance of the filtrate was measured against a blank of 96% methanol on wavelengths of 666 (A_666_) and 653 (A_653_) nm for chlorophyll a and b, respectively, and 470 (A_470_) nm for carotenoids. The results were expressed as mg g^−1^ fresh weight (mg g^−1^ FW) when calculated using the following formulas [Equation (2), Equation (3), Equation (4)] according to Dere et al. [38]:

Chl. *a* content = (15.65 × A_666_) − (7.34 × A_653_) (2)

Chl. *b* content = (27.05 × A_653_) − (11.21 × A_666_) (3)

(4) $$\mathrm{Total}\;\mathrm{carotenoids}\;\mathrm{content}=\frac{1000\times \;A470-\left(2.86\times \;\mathrm{Chl}.a+129.2\times \;\mathrm{Chl}.b\right)}{245}$$

#### 2.3.2. Total Soluble Phenolic Compounds

The total soluble phenolic compounds were determined using Folin–Ciocalteu reagent (FCR) as described previously [39], with slight modification. Briefly, phenolics were extracted from 100 mg of fresh tomato leaves using 20 mL of methanol 80% for 24 h. Subsequently, one mL of FCR (10%) was added to 0.2 mL of methanolic extract of fresh tomato leaves and vortexed for 30 s. Three minutes later, 0.8 mL of 7.5% sodium carbonates (*w*/*v*) was added to the mixture. Subsequently, the mixture was shaken, incubated at room temperature for 30 min, and the absorption was measured at 765 nm. The total soluble phenolics content was expressed as mg gallic acid equivalents per gram fresh weight (mg GAE g^−1^ FW).

#### 2.3.3. Total Soluble Flavonoids

The total soluble flavonoids were determined according to the method of Djeridane et al., 2006 [40]. In brief, one mL of methanolic extract of tomato leaves was mixed with 1 mL of aluminum chloride (2% in methanol). The mixture was strongly shaken and incubated for 15 min at room temperature, and then the absorption was measured at 430 nm. Flavonoid concentration was expressed as mg rutin equivalents per gram fresh weight (mg RE g^−1^ FW)

#### 2.3.4. In Situ Histochemical Localization of O_2_^•−^ and H_2_O_2_

Three terminal leaflets were appraised from each plant from three different positions (top, middle, and lower section), and six biological replicates per treatment were sampled for in situ histochemical localization of superoxide anion (O_2_^•−^) and hydrogen peroxide (H_2_O_2_) using nitro blue tetrazolium (NBT; Sigma–Aldrich, Darmstadt, Germany) and 3,3′-Diaminobenzidine (DAB; Sigma-Aldrich, Darmstadt, Germany), respectively. For O_2_^•−^ histochemical staining, fresh leaflets were vacuum infiltrated, with 10 mM potassium phosphate buffer (pH 7.8) containing 0.1% NBT (*w*/*v*) [41], incubated for 20 min under light and then cleared with 0.15% trichloroacetic acid (*w*/*v*) in ethanol to chloroform 4:1 (*v*/*v*). O_2_^•−^ was visualized as a purple coloration of NBT. For histochemical detection of H_2_O_2_, fresh leaflets were vacuum infiltrated with 0.1% DAB in 10 mM tris buffer (pH 7.8) and then incubated under light for one hour. Following staining, leaflets were cleared as described above and the intensity of brown color was estimated using the ImageJ image processing program (Fiji version; http://fiji.sc; access date 10 May 2021). A Chemi Imager 4000 digital imaging system (Alpha Innotech Corp., San Leandro, CA, USA) was used to measure the discoloration due to NBT or DAB staining.

#### 2.3.5. Antioxidant Enzymes Activity

The enzymatic activities of three antioxidant enzymes including catalase (CAT), guaiacol-dependent peroxidases (POX), and polyphenol oxidase (PPO) were colorimetrically determined at 25 °C using a UV-160 spectrophotometer (Shimadzu, Japan). Briefly, 0.5 g of tomato leaf tissues were homogenized in a pre-freezed mortar and pestle (0–4 °C) using 3 mL of 50 mM Tris buffer (pH 7.8) containing 1 mM EDTA-Na_2_ and 7.5% polyvinylpyrrolidone (PVP). Subsequently, the homogenate was centrifuged for 20 min at 11,269× *g*, at 4 °C. The CAT activity was determined according to [42] on a reaction mixture contained 2 mL of 0.1 M sodium phosphate buffer (pH 6.5), 0.1 mL of 269 mM H_2_O_2_ solution (at a final concentration of 12.5 mM), and 50 μL of crude enzyme extract. The CAT activity was measured by following the decomposition of H_2_O_2_ at 240 nm in a quartz cuvette (extinction coefficient of H_2_O_2_ was 0.040 mM^−1^ cm^−1^).

The POX activity was assessed by measuring the formation of the guaiacol-bound product at 436 nm [43]. The reaction mixture contained 2.2 mL of 100 mM sodium phosphate buffer (pH 6.0), 100 μL guaiacol, 100 μL of 12 mM H_2_O_2_, and 10 μL of crude enzyme extract. The increase in the absorption at 436 nm (A_436_) was measured as the conjugate was formed using an extinction coefficient of 26.6 mM^−1^ cm^−1^ for the conjugate.

The PPO activity was determined according to the method of [44]. Reaction mixture contained 3 mL buffered catechol solution (0.01 M), freshly prepared in 0.1 M phosphate buffer (pH 6.0). The reaction was started by adding 100 µL of crude enzyme extract. Changes in the absorbance at 495 nm (A_495_) were recorded every 30 s for 3 min.

### 2.4. Quantification of Salicylic Acid and Its Precursors Using GC-MS

To better understand the physiological and molecular mechanisms of the defensive role of BA and its derivatives, we examined their effect on salicylic acid (SA) and its biosynthetic associated compounds (_L_-phenylalanine, trans-cinnamic acid [tCA], and BA) using a targeted gas chromatography–mass spectrometry (GC-MS)-based method. Briefly, tomato leaf metabolites were tripartitely extracted using 750 μL of acidic methanol (80%) as described previously [45,46,47]. Subsequently, the supernatants were concentrated under a nitrogen stream to 200 μL, then derivatized with methyl chloroformate (MCF) and analyzed using GC-MS running in the selective ion monitoring (SIM) mode [45,46,47].

Targeted metabolites were examined using the Perkin Elmer Clarus 580 GC with Clarus 560S MS system (Perkin Elmer, Waltham, MA, USA) fitted with Elite-5MS capillary column (crosslinked 5% diphenyl/95% dimethyl polysiloxane stationary phase, 30.0 m length, 0.25 mm inner diameter, 0.25 μm film thickness: Perkin Elmer, Waltham, MA, USA). Helium was used as a carrier gas (1 mL·min^−1^ flow rate). The GC thermo-program was as described previously [45,46,47]. Collected chromatograms were analyzed using TurboMass software (Perkin Elmer, Waltham, MA, USA). _L_-Phenylalanine, tCA, BA, and SA were firstly identified by comparing their mass spectra with library entries of the 2008 version of the NIST/EPA/NIH Mass Spectral Library (NIST 08) and an Automated Mass Spectral Deconvolution and Identification System for GC/MS (NIST-AMDIS) associated with a database of retention index values (National Institute of Standards and Technology, Gaithersburg, MA, USA). Subsequently, the four targeted compounds were confirmed by comparing their retention times (RT), linear retention index (LRI), and mass spectra to authentic standards.

### 2.5. Gene Expression Analysis

The expression levels of 20 antioxidant and SA-associated genes (Table S1) were analyzed in total RNA extracted from tomato leaves (72 h post-treatment [hpt]) using TriZol^®^ reagent (Ambion^®^, Life Technologies, Grand Island, NY, USA). The investigated genes included (i) four antioxidant enzymes included cytosolic ascorbate peroxidase 1 (*SlAPX1*), cytosolic ascorbate peroxidase 2 (*SlAPX2*), superoxide dismutase [Cu-Zn] 1 (*SlCuSOD1*), and iron superoxide dismutase (*SlFeSOD*); (ii) eight salicylic acid biosynthetic genes included one isochorismate synthase (*SlICS*), two aldehyde oxidase (*SlAO1* and *SlAO2*), and five phenylalanine ammonia-lyase (*SlPAL1*, *SlPAL2*, *SlPAL3*, *SlPAL5*, and *SlPAL6*); and (iii) eight pathogenesis-related proteins (PR) included *SlPR*-1, *SlPR1a2*, *SlPRB1*-2, *SlPR4*, *SlPR5*, and *SlPR6*, nonexpressor of pathogenesis-related protein 1 (SlNPR1), and salicylic acid-binding protein (*SlSABP2*). Gene expression was analyzed in duplicate for five biological replicates for each treatment (*n* = 10) as described by [47,48,49,50]. The relative expression was calculated using the 2^−ΔΔ^*^C^*_T_ method [51]. Two housekeeping genes were used for the normalization of gene expression including actin **(***SlACT*) and F-box/kelch-repeat protein (*SlF-box*).

### 2.6. Statistical Analysis

Throughout the study, all experiments were repeated twice (with six biological replicates for each treatment), during two different growing seasons (2020 and 2021), to test the reproducibility and consistency of our findings. Results showed high reproducibility and the values of each pair of repeated experiments were very close to each other. Therefore, only data from the first experiment were analyzed and presented. Data from each pair of repeated experiments were not combined. The data from the repeated experiment did not use in the statistical analysis to avoid the possibility of pseudo-replication. A completely randomized design, with five treatments (six biological replicates per treatment), was used in all experiments (in vitro and in vivo) throughout the study. For the in vitro experiments, probit regression analysis was used to calculate the half-maximal inhibitory concentration (IC50) and IC99 with 95% confidence intervals [33]. Moreover, the analysis of variance (ANOVA), followed by post hoc pairwise comparisons using the Tukey honestly significant difference test (HSD; *p* ≤ 0.05) was used to compare the growth inhibition (%) of each compound at different concentrations. For in vivo study, ANOVA was used to compare between treatments, followed by HSD as a post hoc pairwise comparisons (*p* ≤ 0.05).

## 3. Results

### 3.1. In Vitro Antifungal Activity of Benzoic Acid and Its Hydroxylated Derivatives

**Figure 2:**
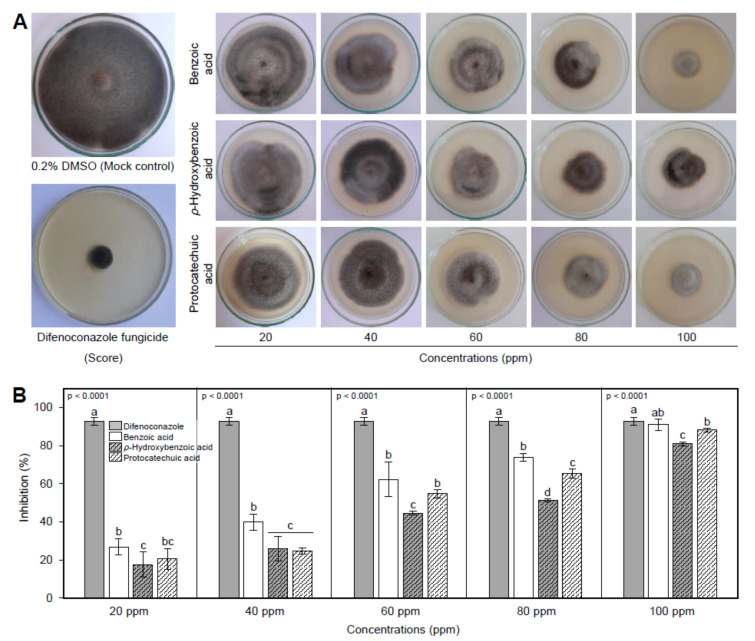
In vitro antifungal activity of benzoic acid (BA) and its hydroxylated derivatives (*ρ*-hydroxybenzoic acid and protocatechuic acid) against *Alternaria solani*. (**A**) Antifungal activity of BAand its hydroxylated derivatives *A. solani*. (**B**) Mycelial radial growth inhibition (%) of *A. solani* after the treatment with BA and its hydroxylated derivatives. Values represent the means ± standard deviation (means ± SD) of six biological replicates (*n* = 6). Different letters indicate statistically significant differences between treatments (*p* < 0.05).

The in vitro antifungal activity of Benzoic acid (BA) and two of its hydroxylated derivatives, *ρ*-hydroxybenzoic acid (HBA), and protocatechuic acid (PCA), showed that all tested compounds showed strong concentration-dependent fungistatic activity against *A. solani* (Figure 2A). The inhibition (%) of mycelial radial growth of *A. solani* was dose-dependent since the higher concentrations showed a wider inhibition zone and vice versa (Figure 2A). At the highest concentration (100 ppm) and just behind the positive control (fungicide), BA had the highest mycelial growth inhibition (90.9 ± 2.9%), followed by PCA (88.1 ± 1.0%) and HBA (80.9 ± 1.0). The same profile was observed at the second two highest concentrations (80 and 60 ppm). It is worth noting that the treatment with 100 ppm BA was comparable to difenoconazole fungicide (92.8 ± 2.1%) with no significant difference between them suggesting similar effectiveness (Figure 2B).

**Figure 3:**
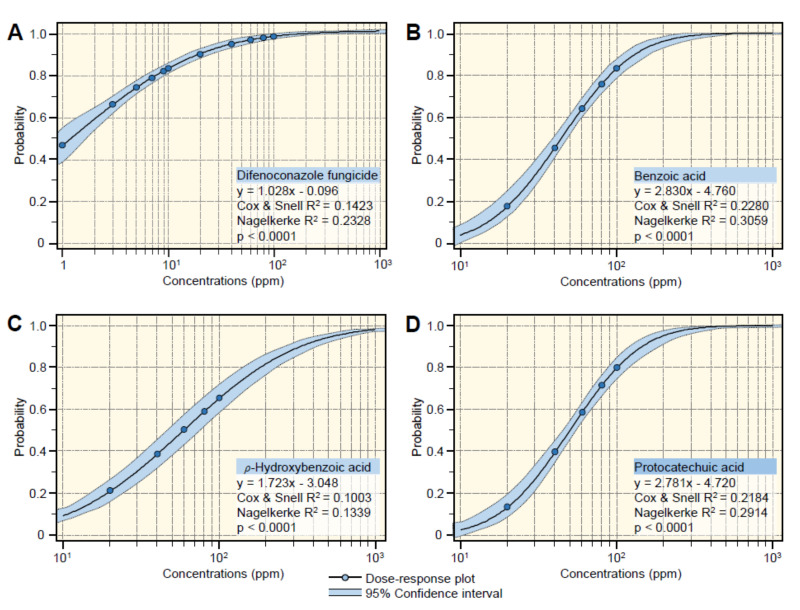
Probit regression analysis of the inhibition effects of benzoic acid (BA) and its hydroxylated derivatives (*ρ*-hydroxybenzoic acid and protocatechuic acid) against *Alternaria solani*. (**A**) The positive control (difenoconazole fungicide), (**B**) benzoic acid, (**C**) *ρ*-hydroxybenzoic acid, and (**D**) protocatechuic acid. Blue dots present the mean of six replicates (*n* = 6). The dose-response regression lines are presented as black solid lines. The 95% CI (confidence intervals) for the estimated regression are light-blue-shaded and edged with dotted lines. Regression equations, Cox, and Snell R2, Nagelkerke R2, and *p*-value based on the F test (*p* < 0.05) were also obtained and presented within the graphs. The experiment was repeated twice with similar results.

According to slope values of probit regression lines (Figure 3), BA and its hydroxylated derivatives, HBA and PCA displayed the same positive upward trend. Regardless the difenoconazole fungicide (Figure 3A), BA had the highest slope value (y = 2.830x − 4.760, Cox and Snell R^2^ = 0.2280, Nagelkerke R^2^ = 0.3059, and *p* < 0.0001) (Figure 3B), followed by PCA (y = 2.781x − 4.720, Cox and Snell R^2^ = 0.2184, Nagelkerke R^2^ = 0.2914, and *p* < 0.0001) (Figure 3C) and HBA which had the lowest slope value (y = 1.723x − 3.048, Cox & Snell R^2^ = 0.1003, Nagelkerke R^2^ = 0.1339, and *p* < 0.0001) (Figure 3D). In agreement with probit analysis, the calculation of the half-maximal inhibitory concentration (IC_50_) and IC_99_ of BA and its hydroxylated derivatives (Table 1) showed that BA had the lowest IC_50_ and IC_99_ (44.69 and 296.60 ppm, respectively), followed by PCA (49.79 and 341.62 ppm, respectively) and HBA (58.80 and 1317.74 ppm, respectively) (Table 1). Collectively, these findings suggest that BA and its hydroxylated derivatives have potent antifungal activity against *A. solani.*

### 3.2. Benzoic Acid and Its Derivatives Reduced the Disease Evaluation of Early Blight

**Figure 4:**
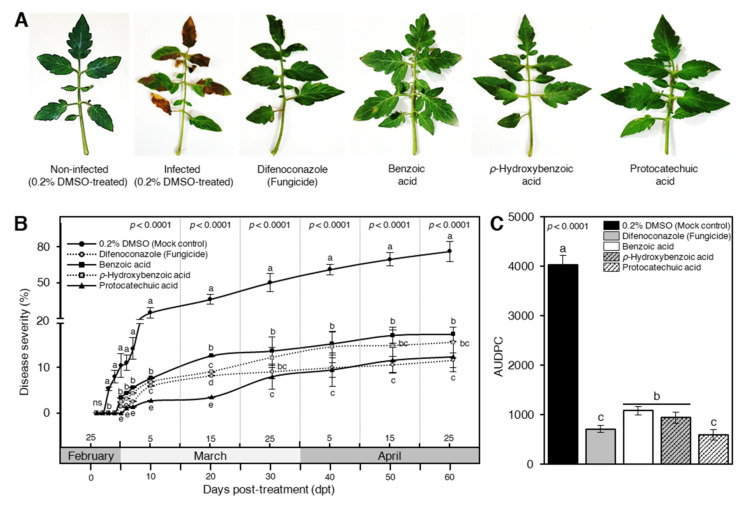
Benzoic acid and its hydroxylated derivatives, ρ-hydroxybenzoic acid (HBA) and protocatechuic acid (PCA), decrease the disease evaluation of early blight caused on tomatoes by *Alternaria solani* under greenhouse conditions. (**A**) Typical symptoms of early blight disease on tomato leaves at 7 days post-treatment (dpt) with 100 ppm of BA or one of its hydroxylated derivatives. (**B**) Disease progress curves of early blight disease on tomato leaves after the treatment with BA or one of its hydroxylated derivatives. (**C**) The area under disease progress curve (AUDPC) of early blight disease on tomato leaves after the treatment with BA or one of its hydroxylated derivatives. Values represent the means ± standard deviation (means ± SD) of six biological replicates (*n* = 6). Different letters indicate statistically significant differences between treatments (*p* < 0.05).

In general, exogenous application with BA and its hydroxylated derivatives significantly reduced the symptoms of early blight on tomato leaves, compared with the non-treated infected plants, at 7 dpt (Figure 4A). Briefly, even though a progressive surge was noticed in the disease severity (%) on the sterilized water (mock)-treated tomato plants throughout the experiment, BA, PHB and, PCA significantly decreased the disease severity (%) at 3 dpt and till the end of the experiment (Figure 4B). PCA was the most effective compound and had the lowest disease severity (%) at 10 and 20 dpt particularly, and throughout the experiment in general, and it was comparable with the positive control (difenoconazole fungicide) (Figure 4B). Likewise, exogenous application with phenolic acids significantly lessened the AUDPC (Figure 4C). PCA-treated plants had the lowest AUDPC value (591.8 ± 102.1) which was comparable to fungicide-treated plants (709.0 ± 69.4). It is worth mentioning that HBA and BA significantly reduced the AUDPC (938.0 ± 113.8 and 1081.6 ± 80.9, respectively) compared with 0.2% DMSO-treated control (4024.8 ± 189.6) (Figure 4C). Collectively, these findings suggest that exogenous application with BA and its hydroxylated derivatives diminish the development of early blight disease and eases the damaging effects of *A. solani* on tomato leaves.

### 3.3. BA and Its Hydroxylated Derivatives Improve the Growth Variables of A. solani-Infected Tomato Plants

**Figure 5:**
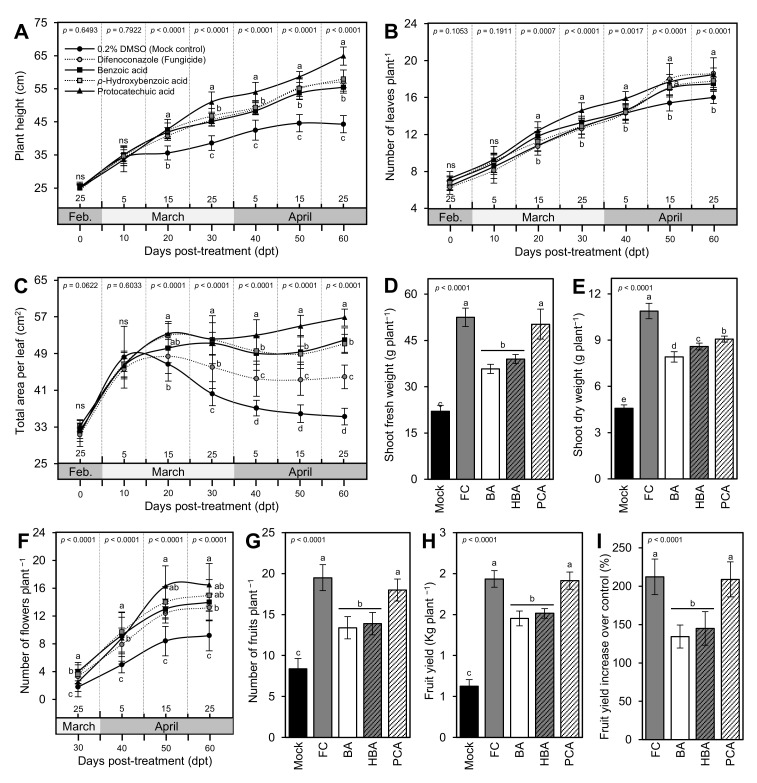
Benzoic acid (BA) and its hydroxylated derivatives, *ρ*-hydroxybenzoic acid (HBA) and protocatechuic acid (PCA), improve the growth variables and enhance fruit yield and its components of *Alternaria solani*-infected tomato plants under greenhouse conditions. (**A**) Plant height (cm), (**B**) number of leaves plant^−1^, (**C**) total area per leaf (cm^2^), (**D**) shoot fresh weight (g plant^−1^), (**E**) shoot dry weight (g plant^−1^), (**F**) number of flowers plant ^−1^, (**G**) number of fruits plant ^−1^, (**H**) fruit yield (Kg plant ^−1^), and (**I**) fruit yield increase over control (%). Values represent the means ± standard deviation (means ± SD) of six biological replicates (*n* = 6). Different letters indicate statistically significant differences between treatments (*p* < 0.05). Mock: 0.2% dimethyl sulfoxide (DMSO)-treated (mock control), FC: difenoconazole-treated (fungicide), BA: benzoic acid-treated, HBA: *ρ*-hydroxybenzoic acid-treated, and PCA: protocatechuic acid-treated.

Starting from 20 dpt, the exogenous application with BA and its hydroxylated derivatives significantly increased the plant height (Figure 5A), the total number of leaves per plant (Figure 5B), and total leaf area (Figure 5C) throughout the experiment, with superiority for PCA treatment. Likewise, the exogenous application with BA and its hydroxylated derivatives considerably heightened both shoot fresh and dry weight per plant (Figure 5D,E, respectively) compared with the mock-treated control plants. Regardless of the fungicide-treated tomato plants, PCA-treated plants had the highest fresh and dry weight, followed by HBA and BA treatments, respectively, which all were significantly higher than the 0.2 DMSO-treated control. Collectively, these findings suggest that the exogenous application of BA and its hydroxylated derivatives has no phytotoxic effect on the treated tomato plants.

### 3.4. BA and Its Derivatives Enhance Fruit Yield and Its Components of Infected Tomato Plants

Generally, exogenous application of BA and its hydroxylated derivatives drastically strengthened all yield components of *A. solani*-infected tomato plants including the total number of flowers per plant (Figure 5F), the number of marketable fruits per plant (Figure 5G), the total weight of fruit yield per plant (Figure 5H), and fruit yield increase over the mock control (Figure 5I) compared with the mock-treated control plants. In terms of all yield components, PCA had the highest number of flowers at 60 dpt (16.50 ± 2.55 flowers plant^−1^), the number of fruits (18.00 ± 1.33 fruits plant^−1^), and fruit yield (2.19 ± 0.11 kg plant^−1^) with an average increase over the mock-treated control of 208.96 ± 22.73% without any significant differences compared to the fungicide-treated control (13.20 ± 1.99 flowers plant^−1^, 19.50 ± 1.58 fruits plant^−1^, 1.94 ± 0.10 kg plant^−1^, and 212.24 ± 23.14%, respectively).

### 3.5. Effect of BA and Its Derivatives on Photosynthetic Pigments of Infected Leaves

**Figure 6:**
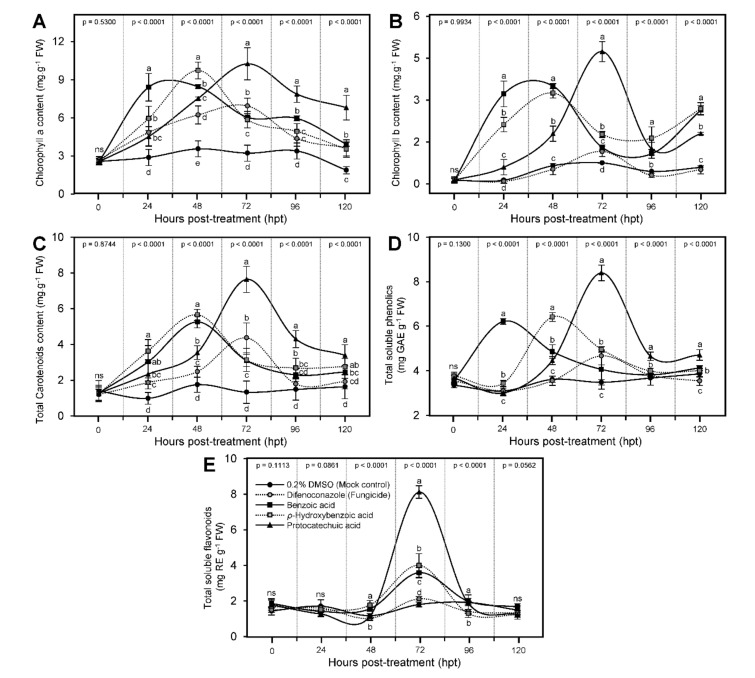
Benzoic acid (BA) and its hydroxylated derivatives, *ρ*-hydroxybenzoic acid (HBA) and protocatechuic acid (PCA), enrich the biochemical profile of *Alternaria solani*-infected tomato plants under greenhouse conditions. (**A**) Chlorophyll *a* content (mg g^−1^ FW), (**B**) chlorophyll *b* content (mg g^−1^ FW), (**C**) total carotenoids content (mg g^−1^ FW), (**D**) total soluble phenolics (mg GAE g^−1^ FW), and (**E**) total soluble flavonoids (mg RE g^−1^ FW). Values represent the means ± standard deviation (means ± SD) of six biological replicates (*n* = 6). Different letters indicate statistically significant differences between treatments (*p* < 0.05).

Generally, the content of chlorophyll a (Figure 6A), chlorophyll b (Figure 6B), and total carotenoids (Figure 6C) were kept higher after the application of BA and its hydroxylated derivatives. It is worth noting that the treatment with BA enhanced the profile of all photosynthetic pigments (chlorophyll *a*, chlorophyll *b*, and total carotenoids) and reached its highest peak 24 hpt (except for total carotenoids where it peaked at 48 hpt) then plummeted again at 72 hpt, whereas the treatment with HBA reached its highest peak 48 hpt and dropped again at 96 hpt (Figure 6). Noteworthy, treatment with PCA reached its highest peak 72 hpt and recorded the highest content of chlorophyll a (10.25 ± 1.26 mg g^−1^ FW), chlorophyll b (4.74 ± 0.36 mg g^−1^ FW), and total carotenoids (7.64 ± 0.73 mg g^−1^ FW) compared with all other treatments and even the fungicide-treated plants (6.94 ± 0.61, 1.16 ± 0.18, and 4.36 ± 0.83 mg g^−1^ FW, respectively). Together, these findings suggest that hydroxylation of BA might delay its physiological role at the biochemical levels.

### 3.6. Benzoic Acid and Its Derivatives Enhanced the Profile of Total Soluble Phenolics and Flavonoids of A. solani-Infected Tomato Plants

Similar to the profile of photosynthetic pigments, all tested compounds enhanced the profile of total soluble phenolics of infected leaves (Figure 6D). Briefly, BA enhanced the profile of total soluble phenolics and reached its highest peak at 24 hpt (6.21 ± 0.13 mg GAE g^−1^ FW) then fell again at 48 hpt, whereas the treatment with HBA reached its highest peak of 48 hpt (6.41 ± 0.20 mg GAE g^−1^ FW) then declined again at 72 hpt. Finally, treatment with PCA reached its highest peak 72 hpt, and recorded the highest content of total soluble phenolics among all treatments (8.40 ± 0.35 mg GAE g^−1^ FW) (Figure 6D). These findings support our hypothesis that hydroxylation of BA might delay its physiological activity at the biochemical levels. On the other hand, the total soluble flavonoid content did not show any positive response during the first 24 hpt. However, it dramatically increased to reach its highest peak at 72 hpt (8.12 ± 0.35, 4.00 ± 0.66, and 3.59 ± 0.29 mg RE g^−1^ FW for PCA, HBA, and BA, respectively) but it dropped thereafter when measured at 96 hpt (Figure 6E).

### 3.7. BA and Its Derivatives Alleviate the Oxidative Stress of A. solani-Infected Leaves

**Figure 7:**
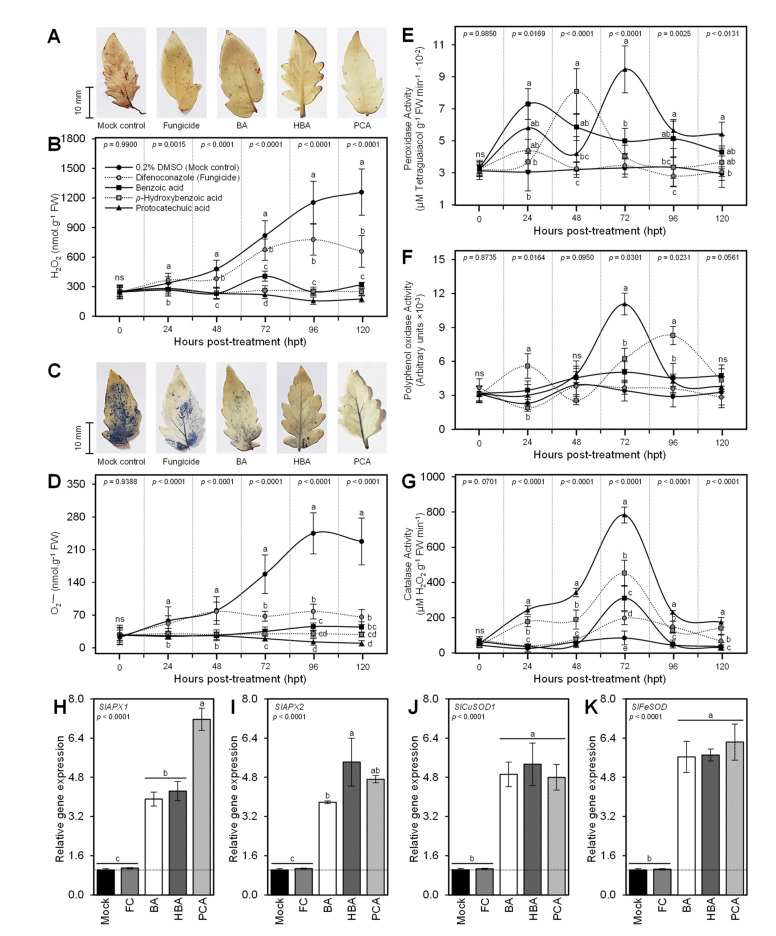
Benzoic acid (BA) and its hydroxylated derivatives, *ρ*-hydroxybenzoic acid (HBA) and protocatechuic acid (PCA), alleviate oxidative stress and enhance the antioxidant defense-related enzymes in *Alternaria solani*-infected leaves under greenhouse conditions. (**A**) In situ histochemical localization of hydrogen peroxide (H_2_O_2_) using DAB-based staining at 72 hpt with BA or one of its hydroxylated derivatives, (**B**) H_2_O_2_ content (nmol g^−1^ FW) after the treatment with BA or one of its hydroxylated derivatives, (**C**) in situ histochemical visualization of superoxide anion (O_2_*^•−^*) using NBT-based staining at 72 hpt with BA or one of its hydroxylated derivatives, (**D**) O_2_*^•−^* content (nmol g^−1^ FW) after the treatment with BA or one of its hydroxylated derivatives, (**E**) peroxidase activity (μM Tetraguaiacol g^−1^ FW min^−1^), (**F**) polyphenol oxidase activity (Arbitrary units), and (**G**) catalase activity (μM H_2_O_2_ g^−1^ FW min^−1^). Values represent the means of six biological replicates (*n = 6*), while whiskers reflect the standard deviation (means ± SD). (**H**–**K**) Relative gene expression of cytosolic ascorbate peroxidase 1 (*SlAPX1*), cytosolic ascorbate peroxidase 2 (*SlAPX2*), superoxide dismutase [Cu-Zn] 1 (*SlCuSOD1*), and iron superoxide dismutase (*SlFeSOD*), respectively. Values represent the means ± standard deviation (means ± SD) of five biological replicates (*n = 5*). Different letters indicate statistically significant differences between treatments (*p* < 0.05). Mock: 0.2% dimethyl sulfoxide (DMSO)-treated (mock control), FC: difenoconazole-treated (fungicide), BA: benzoic acid-treated, HBA: *ρ*-hydroxybenzoic acid-treated, and PCA: protocatechuic acid-treated.

DAB-based in situ histochemical localization of H_2_O_2_ showed that exogenous application of BA and its hydroxylated derivatives notably reduced the accumulation of H_2_O_2_ within the *A. solani*-infected leaves (Figure 7A). Although the mock-treated plants exhibited a progressive accumulation of H_2_O_2_ throughout 120 hpt, all tested compounds significantly reduced and flatten the profile of accumulated H_2_O_2_ (Figure 7B). Likewise, in situ histochemical visualization of O_2_*^•−^* using NBT-based staining showed that the 0.2 DMSO-treated leaves accumulated more blue color (an indicator of O_2_*^•^*^−^) than treated ones (Figure 7C). Exogenous treatment with BA and its hydroxylated derivatives significantly reduced the accumulation of O_2_*^•^*^−^ compared with the 0.2 DMSO-treated control that showed progressive time-dependent accumulation of O_2_*^•^*^−^ (Figure 7D). Taken together, these findings suggest that exogenous treatment with BA and its hydroxylated derivatives alleviate the oxidative stress in *A. solani*-infected tomato leaves.

To better understand how BA and its hydroxylated derivatives alleviate the oxidative stress in infected leaves, we investigated the enzymatic activities of three antioxidant enzymes included guaiacol-dependent peroxidases (POX; Figure 7E), polyphenol oxidase (PPO; Figure 7F), and catalase (CAT; Figure 7G). Briefly, the enzymatic activities of the three enzymes fluctuated after the exogenous application of BA and its hydroxylated derivatives. POX activity dramatically increased 1 dpt with BA (7.30 ± 0.96 × 10^−2^ μM tetraguaiacol g^−1^ FW min^−1^), however, it took 48 hpt with HBA to reach its highest peak (8.09 ± 1.43 × 10^−2^ μM Tetraguaiacol g^−1^ FW min^−1^). Furthermore, treatment with PCA reached its highest peak 72 hpt and recorded the highest POX activity among all treatments (9.47 ± 1.48 × 10^−2^ μM Tetraguaiacol g^−1^ FW min^−1^) (Figure 7E). On the other hand, the enzymatic activities of PPO and CAT dramatically elevated at 72 hpt with PCA, but not earlier (Figure 7F,G, respectively).

Moreover, we investigated the gene expression of four antioxidant enzymes included cytosolic ascorbate peroxidase 1 (*SlAPX1*; Figure 7H), cytosolic ascorbate peroxidase 2 (*SlAPX2*; Figure 7I), superoxide dismutase [Cu-Zn] 1 (*SlCuSOD1*; Figure 7J), and iron superoxide dismutase (*SlFeSOD*; Figure 7K) at 72 hpt. Generally, the four genes (*SlAPX1*, *SlAPX2*, *SlCuSOD1*, and *SlFeSOD*) were significantly upregulated after the treatment with BA and its hydroxylated derivatives compared with both controls. PCA enhanced the expression levels of *SlAPX1* (Figure 7H), whereas HBA induced the relative gene expression of *SlAPX2* (Figure 7I). Likewise, the expressions of *SlCuSOD1* (Figure 7J), and *SlFeSOD* (Figure 7K) were up-regulated in BA and its hydroxylated derivatives-sprayed plants without any significant differences between the three tested compounds.

### 3.8. Benzoic Acid and Its Derivatives Induce the Salicylic Acid Biosynthesis

**Figure 8:**
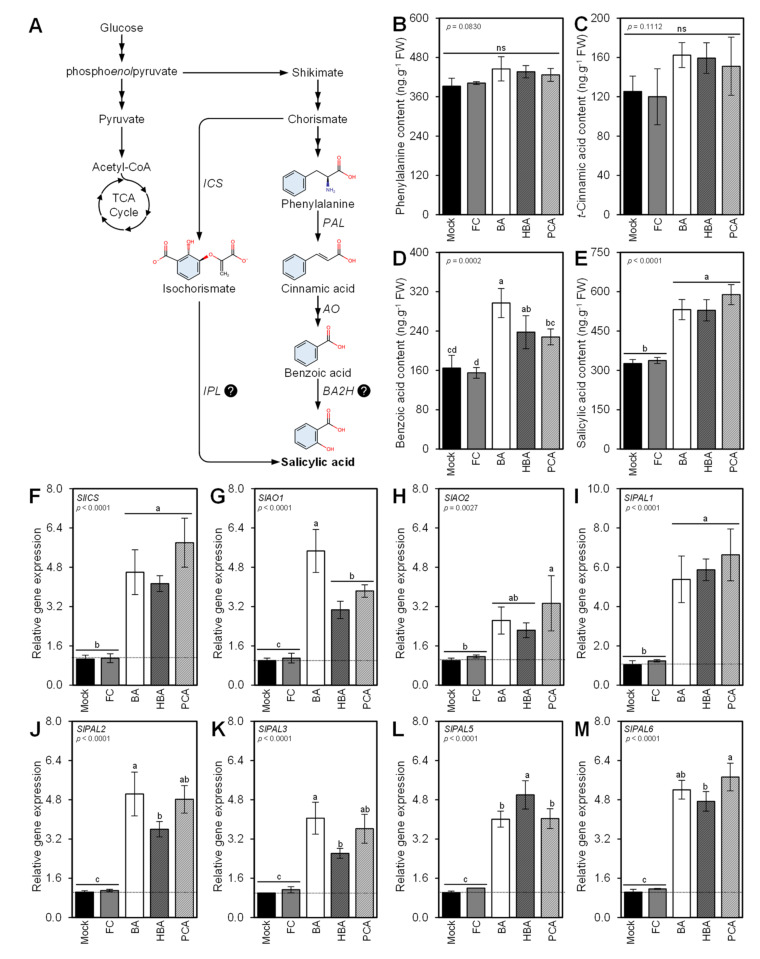
Benzoic acid (BA) and its hydroxylated derivatives, *ρ*-hydroxybenzoic acid (HBA) and protocatechuic acid (PCA), induce the salicylic acid (SA) biosynthesis pathway in *Alternaria solani*-infected leaves under greenhouse conditions. (**A**) Schematic representation of the SA biosynthesis pathway. (**B**–**E**) Endogenous content (ng·g^−1^ FW) of _L_-phenylalanine, *t*-cinnamic acid (*t*CA), BA, and SA, respectively, as detected in tomato leaves after the treatment with BA or one of its hydroxylated derivatives using GC-MS. (**F**) Relative gene expression of isochorismate synthase (*SlICS*), (**G**,**H**) relative gene expression of two aldehyde oxidase (*SlAO1* and *SlAO2*, respectively), and (**I**–**M**) relative gene expression of five phenylalanine ammonia-lyases (*SlPAL1*, *SlPAL2*, *SlPAL3*, *SlPAL5*, and *SlPAL6*, respectively). Values represent the means ± standard deviation (means ± SD) of five biological replicates (*n = 5*). Different letters indicate statistically significant differences between treatments (*p* < 0.05). Mock: 0.2% dimethyl sulfoxide (DMSO)-treated (mock control), FC: difenoconazole-treated (fungicide), BA: benzoic acid-treated, HBA: *ρ*-hydroxybenzoic acid-treated, and PCA: protocatechuic acid-treated.

For a better understanding of the molecular and physiological mechanisms behind the protective role of BA and its hydroxylated derivatives against *A. solani*, we dissected the salicylic acid (SA) biosynthesis pathway (Figure 8A) and its associated genes. The exogenous application of BA and its hydroxylated derivatives did not affect the endogenous levels of early precursors of SA including _L_-phenylalanine (Figure 8B) and *t*-cinnamic acid (Figure 8C). However, it significantly increased the endogenous levels of the intermediate BA with a greater effect for BA (Figure 8D). Likewise, the exogenous application of BA and its hydroxylated derivatives induced the accumulation of SA with no significant differences between tested compounds (Figure 8E). In addition, the expression levels of eight SA biosynthetic genes were significantly increased at 72 hpt with BA and its hydroxylated derivatives. These genes included one isochorismate synthase (*SlICS*; Figure 8F), two Aldehyde oxidases (*SlAO1* and *SlAO2*; Figure 8G,H, respectively), and five phenylalanine ammonia-lyases (*SlPAL1*, *SlPAL2*, *SlPAL3*, *SlPAL5*, and *SlPAL6*) (Figure 8I–M). These findings support our GC-MS findings.

### 3.9. BA and Its Derivatives Stimulate the Expression of Pathogenesis-Related Proteins

**Figure 9:**
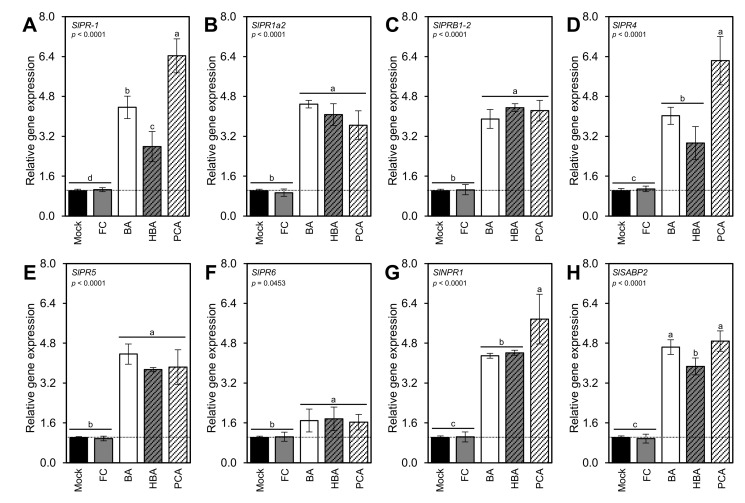
Benzoic acid (BA) and its hydroxylated derivatives, *ρ*-hydroxybenzoic acid (HBA) and protocatechuic acid (PCA), induce the expression of pathogenesis-related proteins in *A. solani*-infected leaves under greenhouse conditions. (**A**–**F**) Relative gene expression of six pathogenesis-related proteins (PR) included *SlPR-1*, *SlPR1a2*, *SlPRB1-2*, *SlPR4*, *SlPR5*, and *SlPR6*, respectively. (**G**) Relative gene expression of nonexpressor of pathogenesis-related protein 1 (*SlNPR1*). (**H**) Relative gene expression of the salicylic acid-binding protein (*SlSABP2*). Values represent the means ± standard deviation (means ± SD) of five biological replicates (*n = 5*). Different letters indicate statistically significant differences between treatments (*p* < 0.05). Mock: 0.2% dimethyl sulfoxide (DMSO)-treated (mock control), FC: difenoconazole-treated (fungicide), BA: benzoic acid-treated, HBA: *ρ*-hydroxybenzoic acid-treated, and PCA: protocatechuic acid-treated.

Along with the induction of SA biosynthesis, exogenous application of BA and its hydroxylated derivatives stimulate the expression levels of eight pathogenesis-related proteins (PR) within 72 hpt. These genes included *SlPR-1* (Figure 9A), *SlPR1a2* (Figure 9B), *SlPRB1-2* (Figure 9C), *SlPR4* (Figure 9D), *SlPR5* (Figure 9E), *SlPR6* (Figure 9F), nonexpressor of pathogenesis-related protein 1 (*SlNPR1*; Figure 9G), and salicylic acid-binding protein (*SlSABP2*; Figure 9H). Although there were no significant differences between the three tested compounds (BA, HBA, and PCA) in terms of *SlPR1a2*, *SlPRB1-*2, SlPR5, and *SlPR6*, PCA greatly induced the transcript levels of *SlPR-*1, *SlPR4*, and *SlNPR1* compared with BA and HBA.

## 4. Discussion

The phenylpropanoid pathway-synthesized phenolic compounds are ubiquitous secondary metabolites found in plants [16,52]. Phenolics play a key role in plant defense and pathogen counter-defense mechanisms against bacteria, fungi, and viruses, and major environmental challenges such as drought and salinity [16,17,53,54,55]. In the current study, we investigated the potential of benzoic acid (BA) and two of its hydroxylated derivatives, ρ-hydroxybenzoic acid (HBA), and protocatechuic acid (PCA) as an alternative management strategy against *A. solani*, the causal agent of early blight on tomato. In vitro studies showed that BA and two of its hydroxylated derivatives exhibited a strong concentration-dependent fungistatic activity against *A. solani.* The high concentrations (100 ppm) of all tested compounds, particularly for BA, effectively inhibited the mycelial growth of *A. solani* and it was almost similar to the positive control (difenoconazole fungicide), without any significant difference between them.

Inhibition of *A. solani* fungal growth was slightly reduced when the hydroxylated derivatives of BA were used. We presume that hydroxylation of BA might reduce the antifungal activity of hydroxylated compounds in a yet unidentified mechanism. It has been reported previously that structural features of BA derivatives conferring their antifungal activity [56]. For instance, stirring the methyl, methoxy, or chloro group on the phenyl ring of BA in the direction *ortho* > *meta* > *para* enhanced their antifungal activity against *A. flavus* and *A. fumigatus* [57]. Likewise, substitution with two or more methyl groups substantially reduced the antifungal activity of BA derivatives against *Cochliobolus lunatus*, *Aspergillus niger*, and *Pleurotus ostreatus* [56]. Collectively, in vitro findings suggest BA and its hydroxylated derivatives have fungistatic action and are able to inhibit the mycelial radial growth of *A. solani* in a dose-dependent manner which exhibited similar potency with the most common fungicide-based control tactic against early blight disease of tomato [58]. Nevertheless, this antifungal activity is associated with the structural features of BA derivatives.

The antifungal activity of BA and its derivatives against *A. solani* might be due to their negative effects on the fungal cell cycle via blocking the cell septation during cell division [59]. Cell septation is an essential process in the fungal cell cycle because it allows the development of new cells, facilitates fungal growth, and regulates mycelia consolidation [60,61]. It worth mention that the *o*-hydroxybenzoic acid (salicylic acid) was previously reported to inhibit the germination of *Penicillium expansum* conidia [62]. Due to the high similarity between SA, BA, and its derivatives structures, we presume the mechanism of action of their antifungal properties are strongly similar. However, this antifungal mechanism is not common, and more microscopic investigations are required to prove this hypothesis.

Another mechanism of action for antifungal activity of BA and its derivatives against *A. solani* might be due to the energy reduction. It is well known that maintenance of the endogenous benzoate levels below the chemical equilibrium value requires energy [63]. For instance, it has been reported that the exogenous application of BA on the ascomycetous yeast, *Zygosaccharomyces bailii*, around the minimum inhibitory concentration (MIC) caused a general energy loss and ATP depletion [63]. Nevertheless, further studies are required to better understand the molecular and cellular mechanisms behind the antifungal roles of BA and its derivatives.

Furthermore, although the disease severity (%) on the mock-treated tomato plants was increased progressively throughout the experiment, BA and its hydroxylated derivatives successfully suppressed the development of early blight symptoms, significantly diminished the disease severity, and reduced the AUDPC on tomato leaves at 7 dpt with no phytotoxicity on the treated plants. Moreover, the exogenous application of BA and its hydroxylated derivatives improves the growth performance, fruit yield, and yield components of *A. solani*-infected tomato plants. This might be due to the reduction of the disease severity; however, more investigations are required to better understand the positive effects of BA and its hydroxylated derivatives on the treated plants.

These findings are in agreement with our previous study where we proofed that the exogenous application of gallic acid and its derivatives efficiently suppressed the development of the disease symptoms and decreased the AUDPC on treated tomato plants [64]. Likewise, exogenous application of BA significantly reduced the disease severity and disease incidence of rice brown spot disease, caused by the ascomycetous fungus *Cochliobolus miyabeanus* (anamorph: *Bipolaris oryzae*) [65], and the cocoa vascular streak dieback, caused by basidiomycetous fungus *Oncobasidium theobromae* (syn. *Thanatephorus theobromae*) [66]. Collectively, these findings suggest that exogenous application with BA and its hydroxylated derivatives diminish the development of early blight disease and eases the damaging effects of *A. solani* on tomato leaves. However, the physiological and biochemical mechanisms behind these roles are poorly understood.

One of the most accepted hypotheses regarding the protective role(s) of BA particularly, and phenolic compounds in general, is that role might be due to the elevated endogenous phenolics content. Herein, we showed that BA and its hydroxylated derivatives enhanced the profile of total soluble phenolics, and flavonoids in treated plants which were negatively correlated with disease progression and could directly impede the colonization of leaf tissues by *A. solani*, in agreement with our previous study using some other phenolic compounds [64]. Recently, it has been reported that the exogenous application of HBA significantly induced root border cells of grapevine seedlings to produce more phenolic acids [67]. Likewise, AUDPC of spot blotch disease in bread wheat, caused by *Bipolaris sorokiniana*, was negatively correlated with pathogen-induced phenolics [68,69]. Together, these findings suggest that the exogenous application of BA and its hydroxylated derivatives suppress the development of the disease symptoms via induction of endogenous phenolics content.

Moreover, in situ histochemical visualization assays showed that infection with *A. solani* induces the accumulation of reactive oxygen species (ROS), particularly H_2_O_2_ and O_2_*^•−^*, which generate strong oxidative stress in infected leaves. However, the exogenous application of BA and its hydroxylated derivatives (HBA and PCA) notably reduced the accumulation of both free radicals in *A. solani*-infected leaves. These findings suggest that BA and its derivatives induce the activation of a multilayered antioxidative system to neutralize the harmful effect of ROS and to maintain their homeostasis within infected leaves. This complex system encompasses two major mechanisms, enzymatic and non-enzymatic antioxidant defense machinery. The enzymatic antioxidant defense machinery serves as the front-line in antioxidant defenses, whereas non-enzymatic antioxidant defense machinery represents the second line of defense against ROS [70,71,72,73].

The enzymatic antioxidant defenses mainly rely on a variety of scavenging enzymes including APX, CAT, GPX, and SOD [71,72]. These enzymes directly scavenge H_2_O_2_ and O_2_*^•−^* and reduce their reactivity [71,73]. Interestingly in our study, the enzymatic activities of several antioxidant enzymes, including POX, PPO, and CAT, were dramatically increased at 72 hpt with BA and its hydroxylated derivatives. Likewise, the relative expression levels of cytosolic ascorbate peroxidase 1 and 2 (*SlAPX1* and *SlAPX2*), superoxide dismutase [Cu-Zn] 1 (*SlCuSOD1*), and iron superoxide dismutase (*SlFeSOD*) were upregulated at 72 hpt. These findings prove that BA and its hydroxylated derivatives play a key role in the activation of enzymatic antioxidant defense machinery to help tomato plants to cope with the harmful oxidative stress caused by *A. solani*.

On the other hand, the non-enzymatic antioxidant defense machinery includes hydrophilic metabolites such as phenolics and flavonoids and lipophilic antioxidants such as carotenoids [70,71]. These antioxidant molecules act as a second line of defense against ROS [70,71,72]. It has been reported that the cellular antioxidant capacity in higher plants is associated with total phenolics content [74]. It is worth mentioning that all these metabolites (phenolics, flavonoids, and carotenoids) were increased markedly at 72 hpt with BA and its hydroxylated derivatives. These findings suggest that BA and its and its hydroxylated derivatives are involved in the activation of the non-enzymatic antioxidant defense machinery as a second line of antioxidant defenses against *A. solani*.

Moreover, the exogenous application of BA and its hydroxylated derivatives induced the accumulation of endogenous BA and SA, but not _L_-phenylalanine and *t*CA. These findings suggest that exogenous BA directly elevates the endogenous BA levels, and subsequently enhances the SA levels. SA is a stress-associated phytohormone that contributes directly to plant defense responses [75]. Briefly, SA plays a key role in defense response against biotrophic and hemibiotrophic phytopathogens and is directly involved in the activation of systemic acquired resistance (SAR) [76,77,78].

In higher plants, SA is synthesized from the shikimate pathway via two different routes which both starting from the intermediate chorismate [79]. The first route for SA biosynthesis starts by conversion of chorismate to isochorismate using isochorismate synthase (*ICS*) [80,81] then to SA by the activity of isochorismate pyruvate lyase (*IPL*) [82]. It has been reported previously that most plant species possess only one or two gene copies of *ICS* [79]. In congruence with this fact, our findings showed that the tomato genome possesses only one *ICS* homolog (*SlICS*; NCBI accession number NP_001234794.1). Interestingly, *SlICS* was upregulated at 72 hpt with BA and its hydroxylated derivatives. On the other hand, there is no evidence for the existence of *IPL* in tomatoes and this enzyme has only been discovered in bacteria [83].

The second route for SA biosynthesis starts with the conversion of _L_-phenylalanine to CA by the activity of *PAL* [82]. Subsequently, CA is converted to BA using aldehyde oxidase (*AO*) then to SA by the activity of benzoic acid-2-hydroxylase (*BA2H*) [84,85]. In the current study, at least two aldehyde oxidases (*SlAO1* and *SlAO2*), and five phenylalanine ammonia-lyases (*SlPAL1*, *SlPAL2*, *SlPAL3*, *SlPAL5*, and *SlPAL6*) were upregulated at 72 hpt with BA and its hydroxylated derivatives. Although *BA2H* was previously purified from tobacco and suggested to be involved in SA biosynthesis [86], the gene encoding this enzyme is not cloned yet and there is no evidence for the existence of *BA2H* gene probes within the tomato genome. The absence of both *IPL* and *BA2H* makes the SA biosynthesis to remain unclear and suggests that BA and its hydroxylated derivatives tomato plants might generate SA from chorismate through an unknown mechanism.

The higher levels of SA were associated with the upregulation of six pathogenesis-related proteins (PR) included *SlPR-1*, *SlPR1a2*, *SlPRB1-2*, *SlPR4*, *SlPR5*, and *SlPR6*, nonexpressor of pathogenesis-related protein 1 (*SlNPR1*), and salicylic acid-binding protein (*SlSABP2*) at 72 hpt with BA and its hydroxylated derivatives. It has been shown previously that SA induced resistance to *A. solani* in hydroponically grown tomato via the induction of PR-1B gene expression which accumulated at 24 hpt and continued to be strongly expressed at the 48 hpt [87]. Likewise, exogenous application with low concentrations (0.01 mM) of methyl salicylate enhanced the resistance of tomato fruit to chilling via the induction of PR-2b, PR-3a, and PR-3b [88].

**Figure 10:**
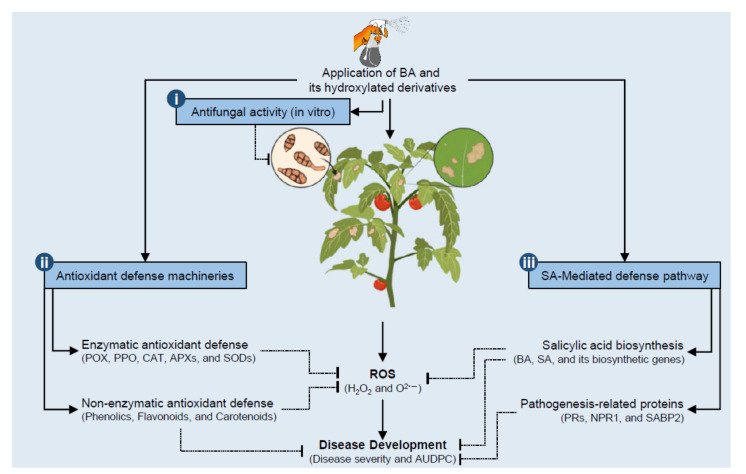
Schematic representation of a hypothetical model for the potential defensive roles of benzoic acid (BA) and its hydroxylated derivatives, *ρ*-hydroxybenzoic acid (HBA) and protocatechuic acid (PCA), in tomato against *A. solani.* Briefly, BA and its hydroxylated derivatives attenuate the harmful effect of *A. solani* on tomato plants via a complex multilayered defense system that includes: (i) BA and its hydroxylated derivatives (HBA and PCA) might have potent antifungal activity against *A. solani.* (ii) BA and its derivatives neutralize and maintain ROS homeostasis within infected leaves via the induction of multilayered antioxidative machinery that includes enzymatic (include *POX*, *PPO*, *CAT*, *APX*s, and *SODs*) and non-enzymatic (phenolics, flavonoids, and carotenoids) antioxidant defenses. (iii) BA and its hydroxylated derivatives induce the SA-mediated pathway, which is implicated in ROS homeostasis and associated with defense response for fungal phytopathogens to reduce disease development (disease severity and AUDPC). The SA-mediated defense relies on two major components. The first component includes SA and its biosynthetic genes (*PALs*, *AOs*, and *ICS*), whereas the second component includes pathogenesis-related proteins (*SlPR-1*, *SlPR1a2*, *SlPRB1-2*, *SlPR4*, *SlPR5*, *SlPR6*), nonexpressor of pathogenesis-related protein 1 (*SlNPR1*), and salicylic acid-binding protein (*SlSABP2*). Solid lines with arrows represent positive reactions, whereas blunt-ended dotted lines indicate negative regulation. For more details and the full names and abbreviations, see the main text.

To summarize these findings, a hypothetical model of the potential roles of BA and its hydroxylated derivatives in tomato defense against *A. solani* is proposed and presented in Figure 10. Briefly, these findings proved that BA and its hydroxylated derivatives attenuate the harmful effect of *A. solani* on tomato plants via a complex multilayered defense system that includes at least three major mechanisms. (i) BA and its hydroxylated derivatives (HBA and PCA) play a strong concentration-dependent fungistatic activity against *A. solani.* (ii) BA and its derivatives induce the activation of a multilayered antioxidative system to neutralize the harmful effect of ROS and to maintain their homeostasis within infected leaves. This antioxidative system relies on two major mechanisms. The enzymatic antioxidant defense machinery (include *POX*, *PPO*, *CAT*, *APX*s, and *SODs*) serves as the front-line in antioxidant defenses, whereas non-enzymatic antioxidant defense machinery (phenolics, flavonoids, and carotenoids) represent the second line of defense against ROS. (iii) BA and its hydroxylated derivatives induce the SA-mediated pathway, which is implicated in ROS homeostasis and associated with defense response against *A. solani* infection. SA-mediated pathway relay on two major components that include SA and its biosynthetic genes (*PALs*, *AOs*, and *ICS*) and pathogenesis-related proteins, nonexpressor of pathogenesis-related protein 1 (*SlNPR1*), and *SABP2*. The findings of this study do not only outline the potential application of BA and its hydroxylated derivatives as a sustainable alternative control strategy against *A. solani* but also deciphering the physiological and biochemical mechanisms behind their protective role.

## Figures and Tables

**Table 1:** The half-maximal inhibitory concentration (IC_50_) and IC_99_ values (ppm) of difenoconazole fungicide, Benzoic acid, and two of its hydroxylated derivatives (*ρ*-hydroxybenzoic acid and protocatechuic acid) against *A*. *solani* (*n* = 6).

Compounds	IC_50_ (ppm)	95% Confidence Interval		IC_99_ (ppm)	95% Confidence Interval		Overall Model Fit		
		Lower	Upper		Lower	Upper	χ^2^	*p*-Value	Cox and Snell *R^2^*
Difenoconazole	1.24	0.81	1.71	226.67	134.61	462.59	168.92	<0.0001	0.1423
Benzoic acid	44.69	40.05	49.31	296.60	223.04	445.77	129.36	<0.0001	0.2280
*ρ*-Hydroxybenzoic acid	58.80	45.96	75.24	1317.74	1015.76	1709.49	52.84	<0.0001	0.1003
Protocatechuic acid	49.79	44.84	54.96	341.62	251.26	532.72	123.18	<0.0001	0.2184

## Data Availability

The data supports the findings of this study are contained within the article or supplementary material and available from the corresponding author upon reasonable request.

## Supplementary Materials

The following are available online at https://www.mdpi.com/article/10.3390/jof7080663/s1, Table S1. Primers used for gene expression analysis of antioxidant enzymes, salicylic acid biosynthesis genes, and pathogenesis-related proteins of tomato (*Solanum lycopersicum*) using real-time RT-PCR.
