# Supplementary material for: Benzoic Acid and Its Hydroxylated Derivatives Suppress Early Blight of Tomato (Alternaria solani) via the Induction of Salicylic Acid Biosynthesis and Enzymatic and Nonenzymatic Antioxidant Defense Machinery
Source: J Fungi (Basel). 2021 Aug 16;7(8):663. doi: 10.3390/jof7080663 (PMC8400885; doi:10.3390/jof7080663)
Supplement: Supplementary file 1 [file jof-07-00663-s001.zip › jof-1310096-supplementary.pdf]

Journal of
Fungi

MDPI

Supplementary Materials:

Table S1. Primers used for gene expression analysis of antioxidant enzymes, salicylic acid biosynthesis genes, and pathogenesis-related proteins of tomato (*Solanum lycopersicum*) using real-time RT-PCR [a].

Description	Gene	NCBI Accession number		Primer (Forward and Reverse)	TM (˚C)	Product size (bp)
**Antioxidant enzymes**						
Cytosolic ascorbate peroxidase 1	*SlAPX1*	NM_001247853.2	F	GGCTCTCCTTTGTGATCCTG	59.80	211
			R	CAAAAACAACAGCTCCAGCA	60.03	
Cytosolic ascorbate peroxidase 2	*SlAPX2*	NM_001247859.2	F	TACGGGAGGACCTGATGTTC	59.93	216
			R	CAAGGTCCCTCAAAACCAGA	60.08	
Superoxide dismutase [Cu-Zn] 1	*SlCuSOD1*	NM_001247102.2	F	TGAATTGGGGTTGAACCATT	60.03	189
			R	GCAGGCACTGTAATCTGCAA	60.02	
Iron superoxide dismutase	*SlFeSOD*	NM_001313769.1	F	GCCACTGCCTCTGCTAATTC	59.98	193
			R	TATGAGGCTCCAAAGCATCC	60.18	
**Salicylic acid biosynthesis genes**						
Isochorismate synthase	*SlICS*	NM_001247865.2	F	CATTGTCGATGAATGGATGC	59.89	243
			R	CTTTGGAGAGTCCGAGCAAC	59.99	
Aldehyde oxidase	*SlAO2*	NM_001247524.1	F	CGGTTTCCATGCTTCTCAAT	60.07	209
			R	CAAAAGTCTTGCAGGCATCA	59.99	
Phenylalanine ammonia-lyase 1	*SlPAL1*	XM_004234584.3	F	ACGGGTTGCCATCTAATCTG	59.96	197
			R	AGCTGTTTTCCTGGCTGAAA	59.99	
Phenylalanine ammonia-lyase 2	*SlPAL2*	NM_001320601.1	F	AGGCTACTCCGGCATAAGGT	60.12	223
			R	AATACCAGCCTGTTGGAACG	59.99	
Phenylalanine ammonia-lyase 3	*SlPAL3*	NM_001320609.1	F	GAAAACCGCTGAGGCTGTAG	60.02	219
			R	CTGTCCACAACTCGAAGCAA	60.02	
Phenylalanine ammonia-lyase 5	*SlPAL5*	NM_001320040.1	F	GCCAAGCTATCGACTTGAGG	59.98	190
			R	CAGGGGTCATCAGCATAGGT	59.95	
Phenylalanine ammonia-lyase 6	*SlPAL6*	XM_004249510.4	F	TTCAGGGGTACTCTGGCATC	60.07	204
			R	CCTCAGCATTCAACGTCTCA	59.98	
**Pathogenesis-related proteins**						

Description	Gene	NCBI Accession number		Primer (Forward and Reverse)	TM (°C)	Product size (bp)
Pathogenesis-related protein 1	*SlPR-1*	XM_004242627.4	F	GGTGGGCAAATTCAAGAAGA	60.05	194
			R	CCCACATAATTGCCCTTCAA	60.69	
Pathogenesis-related protein 1a2	*SlPR1a2*	NM_001321040.2	F	GCTCAAAATTCACCCCAAGA	60.05	162
			R	CTCCCCTGAACCAGAATGAA	60.04	
Pathogenesis-related protein B1-2	*SlPRB1-2*	XM_019216060.1	F	CGGTGAACACTGGAAATGTG	60.00	189
			R	GGAGCATCGCCATTAATCAT	59.89	
Pathogenesis-related leaf protein 4	*SlPR4*	XM_004246675.4	F	GTAACAACGGGTGGTGGTTC	60.13	246
			R	ATGCCCACCCTTCCCTATAC	60.04	
Pathogenesis-related protein 5	*SlPR5*	NM_001330783.1	F	GAGTCCTGGATTGCAAAGGA	60.20	209
			R	AAGTGAACCAGGGCATTCAC	59.97	
Pathogenesis-related leaf protein 6	*SlPR6*	NM_001247385.2	F	TCCGAGAGGCCAAGCTATAA	59.94	189
			R	GTAAGGACGTTGTCCGATCC	59.41	
Nonexpressor of Pathogenesis-related protein 1	*SlNPR1*	NM_001247629.2	F	GCTTTTTCGGATTCCAATGA	60.02	157
			R	CAGCGAAGAAGTCGAAATCC	59.96	
Salicylic Acid Binding Protein	*SlSABP2*	XM_004233374.4	F	AACGGACACCAGCAGAGAAT	59.73	198
			R	TGGCCTTTGACAAATCTTCC	60.05	
**Reference genes**						
Actin	*SlACT*	NM_001330119.1	F	ATGGTGGGTATGGGTCAAAA	60.07	197
			R	GGGGCTTCAGTTAGGAGGAC	59.91	
F-box/kelch-repeat protein	*SlF-box*	XM_004234570.4	F	CGGTTGACCCGATAAGAAAA	59.93	200
			R	GGTCGCGTATCAGGGTAGAA	60.10	

[a] The listed genes were assembled based on recent available data in national center for biotechnology information website (NCBI, http://www.ncbi.nlm.nih.gov/gene/).
